# Pulmonary artery pseudoaneurysm as a neglected manifestation of pulmonary mucormycosis: a retrospective observational study

**DOI:** 10.1186/s12879-026-13304-8

**Published:** 2026-04-21

**Authors:** Bich-Ngoc Nguyen-Thi, Doan Pham-Van, Nguyen Tran-Ngoc, Khoa Nguyen-Dang, Hoang-Anh Nguyen-Cong, Michel Pletschette, Luong Van Dinh

**Affiliations:** 1 National Lung Hospital, Hanoi, Vietnam; 2 School of Medicine and Pharmacy at Hanoi, Vietnam National University, Hanoi, Vietnam; 3 Department of Tuberculosis and Lung Diseases, School of Medicine, University of Medicine and Pharmacy at Ho Chi Minh City, Ho Chi Minh City, Vietnam; 4 Department of Internal Medicine, School of Medicine, University of Medicine and Pharmacy at Ho Chi Minh City, Ho Chi Minh City, Vietnam; 5 Respiratory Department, University Medical Center Ho Chi Minh City, Ho Chi Minh City, Vietnam; 6 Institute of Infectious Diseases and Tropical Medicine, University of Munich LMU Medical Center, Munich, Germany

**Keywords:** Pulmonary artery pseudoaneurysm, Pulmonary mucormycosis, Hemoptysis, Case series, Diabetes

## Abstract

**Introduction:**

Pulmonary artery pseudoaneurysm (PAP) is a rare but potentially fatal vascular abnormality, while its occurrence secondary to pulmonary mucormycosis (PM) is uncommon. The coexistence of PAP and PM represents an exceedingly uncommon and life-threatening condition, with only a limited number of cases reported in the literature.

**Method:**

We retrospectively analyzed medical records of 69 patients diagnosed with PM at the Center of Rare Lung Diseases and Respiratory Infections, National Lung Hospital, Hanoi, Vietnam, between January 2020 and April 2025. Among them, nine patients with concomitant PAP were identified, and detailed data on clinical characteristics, diagnostic evaluations, management, and outcomes were extracted from electronic medical records.

**Results:**

The nine patients had a median age of 61 years (range 51–88), with males predominating and diabetes mellitus documented in eight cases, most of which were poorly controlled. Hemoptysis was mild in the majority, with only one patient developing massive bleeding. Chest CT revealed 16 PAPs (10 round type, 5–40 mm in diameter), with bilateral involvement in over half of the patients. Surrounding abnormalities of PAPs included pulmonary consolidation in all nine cases and necrosis in eight. Management included surgical resection in five patients, and antifungal therapy with amphotericin B followed by triazoles, but three patients died, two from fatal hemoptysis.

**Conclusion:**

Systematic screening for PAP in PM, especially in patients with poorly controlled diabetes and pulmonary consolidation or necrosis, along with early antifungal therapy and timely surgical intervention, may improve survival in this rare but life-threatening condition.

## Background

Pulmonary artery pseudoaneurysm (PAP) represents a rare vascular disorder in which disruption of the arterial wall results in leakage of blood outside the vessel [1, 2]. In contrast to a true aneurysm, the pseudoaneurysmal sac does not consist of all three normal layers of the arterial wall but rather is contained by surrounding tissues or thrombus [1, 2]. Although the condition is not widely reported, existing studies indicate that the prevalence of PAP reaches approximately 2% among patients presenting with hemoptysis, and this figure may rise to up to 11% in those undergoing bronchial angiography [3, 4]. PAP may result from various causes, such as trauma, congenital diseases, malignancies, or connective tissue disorders [5, 6]. Among causes, infection is the most common, typically associated with infective endocarditis, septic emboli, or pyogenic bacteria, whereas PAP due to mucormycosis is rarely reported [3, 5, 7, 8].

Mucormycosis is uncommon among invasive fungal infections, accounting for approximately 1.5% of cases [9]. Pulmonary mucormycosis (PM) represents the third most frequent site of involvement [10]. PM is often challenging to diagnose due to its nonspecific clinical manifestations and imaging features, but it carries a high mortality rate of 72% if left untreated [11, 12]. Hemoptysis is not a mandatory symptom of PM and has been reported in only about one-third of patients [11, 13]. Histopathological evidence has demonstrated that Mucor species possesses angioinvasive potential, enabling invasion of the pulmonary vasculature and subsequent development of PAP [7, 14, 15]. PAP may remain asymptomatic or present with minor hemoptysis, but in severe cases, it can rupture or result in massive, life-threatening hemorrhage and death.

Both PAP in the context of hemoptysis and PM as an invasive fungal infection are infrequent. Therefore, the coexistence of PAP related to PM represents an even more uncommon clinical condition, with only a limited number of reports available to date. We present here a retrospective observational study, to our knowledge, the largest to date, comprising nine patients from Vietnam with confirmed PM complicated by PAP and aimed at highlighting clinical features, diagnostic challenges, and outcomes.

## Methods

### Design and setting of the study

We retrospectively reviewed the medical reports of all 69 patients diagnosed with PM through Electronic Medical Record (EMR) at the Center of Rare Lung Diseases and Respiratory Infections, National Lung Hospital, Hanoi, Vietnam, between January 2020 and April 2025.

### Participants

All patients received a definitive diagnosis of PM based on the gold standard of histopathology of bronchopulmonary tissue and/or ≥ 2 positive cultures for *Mucor* species from respiratory specimens, accompanied by appropriate clinical manifestation and risk factors.

### Materials and data collection

**Fig. 1:**
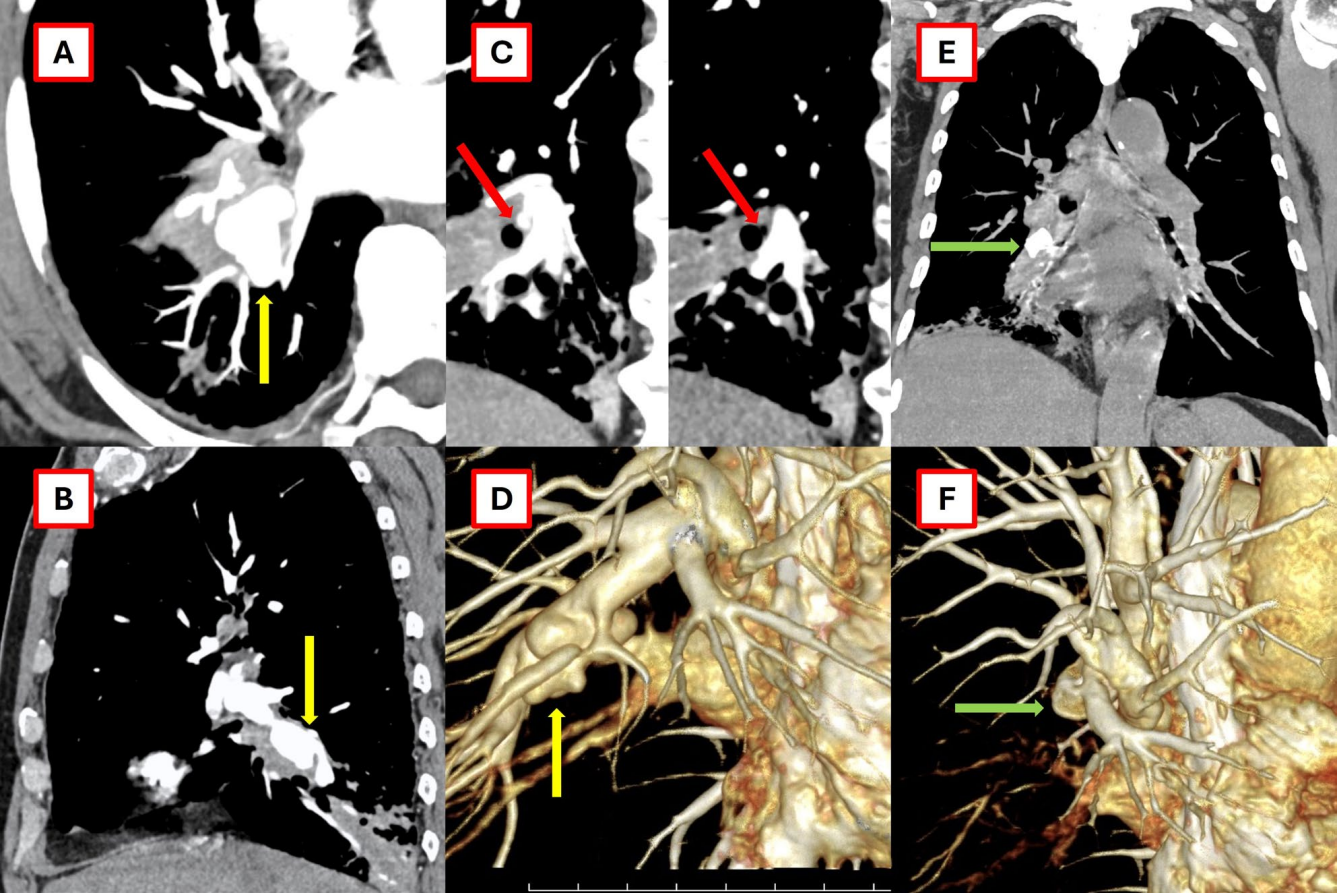
Images of the pulmonary artery pseudoaneurysm (PAP) in the patient (case 1) are shown in axial, sagittal, and 3D views. **A** and **B** demonstrate the PAP in the right intermediate branch of the pulmonary artery (yellow arrow). **C** shows the PAP invading the adjacent bronchus (red arrow). **D** presents a 3D image of the PAP. **E** and **F** describe images taken 2 years after pulmonary artery coil embolization (green arrow)

**Fig. 2:**
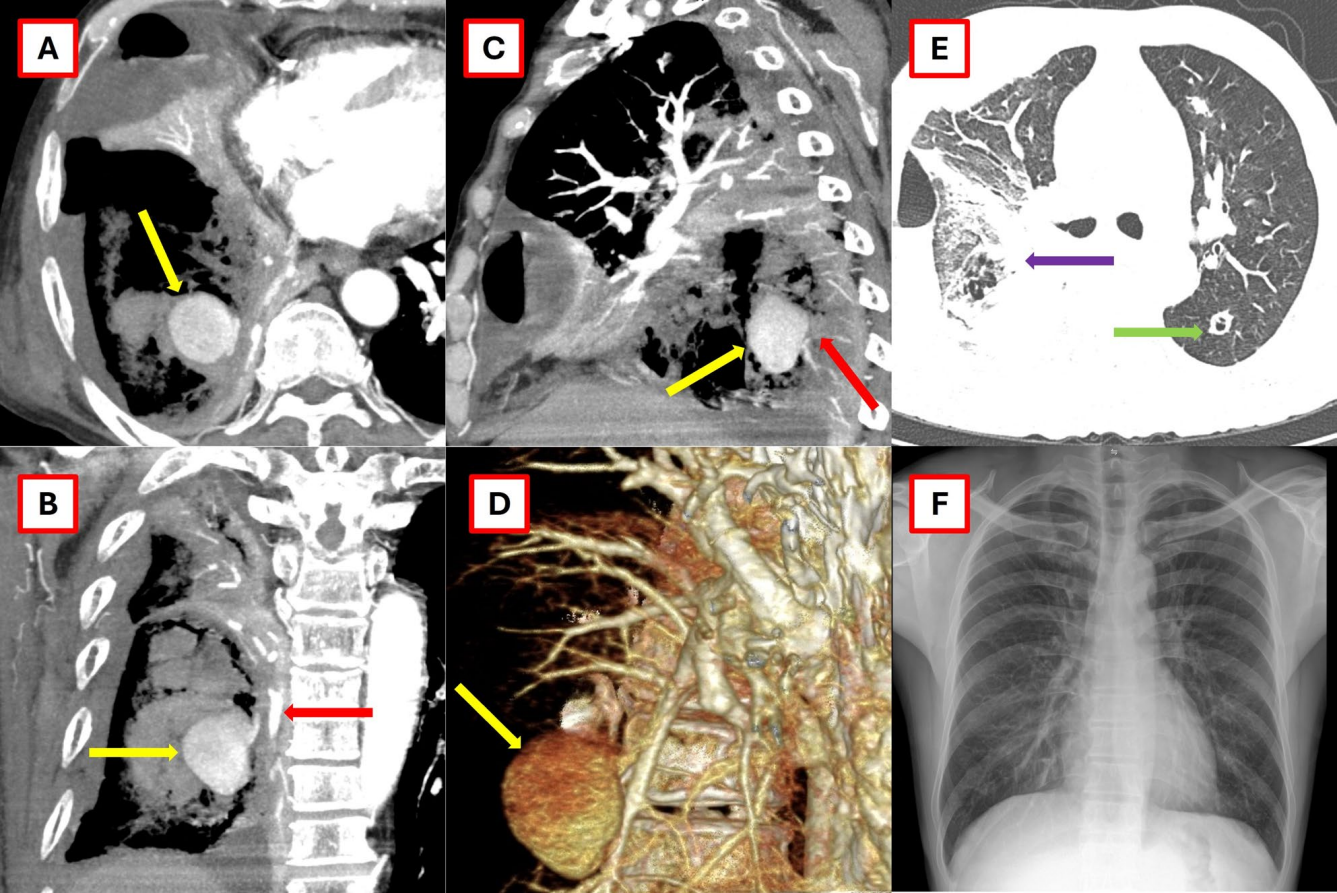
Images of the pulmonary artery pseudoaneurysm (PAP) in the patient (case 3) are shown in axial, sagittal, and 3D views. **A** and **B** demonstrate the PAP in the posterior basal branch of the right pulmonary artery (yellow arrow). **B** and **C** show the PAP invading the adjacent bronchus and pleura (red arrow). **B** and **D** show a suspected systemic-to-pulmonary arterial shunt. **E** depicts the reverse halo sign (purple arrow) and necrotic nodules (green arrow). **F** shows the image 1 month after lobectomy

All chest computed tomography (CT) (contrast-enhanced) scans obtained at the earliest time point following admission were independently reevaluated by two experienced radiologists (B.N.N.T. and N.T.N.) to determine the presence of PAP, with final diagnoses reached by consensus (Figs. 1 and 2). A descriptive observational study was performed on all cases identified with PAP.

We collected data on clinical features, laboratory tests, microbiological findings, bronchoscopy, contrast-enhanced chest CT results, diagnostic approaches for PM, management of hemoptysis, antifungal therapy, and patient outcomes at discharge, all derived from electronic medical records. Comorbidities were documented based on discharge diagnoses. Hemoptysis was classified as massive if exceeding 100 mL/24 hours and mild if less than 100 mL/24 hours [16]. Laboratory test results were recorded at the time of admission. Only the initial sputum and blood culture samples obtained immediately after admission were included in the analysis.

### Ethics approval

The study protocol was reviewed and approved by the Institutional Review Board (IRB) of National Lung Hospital, Hanoi, Vietnam (Approval Number: 121/25/CN-HĐĐĐ-BVPTƯ). Written informed consent for participation was obtained from the patient; in instances where the patient was deceased, consent was obtained from the legally authorized representative.

## Results

Out of 69 patients diagnosed with PM, nine cases (13%) of PM with concomitant PAP were included in this cohort.

Table 1 summarizes the clinical characteristics, laboratory, and microbiological findings of nine patients. The median age was 61 years (range 51–88), with males accounting for the majority. The duration of hospitalization ranged from 5 to 45 days, with diabetes mellitus being the predominant comorbidity observed in eight patients. Massive hemoptysis was observed in a single case, whereas most patients either experienced mild hemoptysis or did not report it. Most patients exhibited poor glycemic control, elevated C-reactive protein levels and white blood cell counts, as well as anemia. No abnormalities in coagulation or platelet counts were recorded (data not shown). The median time from symptom onset to diagnosis of PAP was 32 [20–45] days (median [IQR]).

**Table 1:** Clinical features, blood test results, and microbiological findings of nine patients

Variables	Case 1	Case 2	Case 3	Case 4	Case 5	Case 6	Case 7	Case 8	Case 9
Age, sex	82, male	65, male	56, male	76, male	51, female	64, male	53, female	56, male	88, male
Duration of hospitalisation (days)	20	40	30	5	45	21	23	12	38
Symptom onset to PM Dx (days)	15	30	20	20	40	50	40	25	30
Symptom onset to PAP Dx (days)	45	48	13	17	20	47	32	35	30
ICU admission	Yes	No	No	Yes	No	No	No	No	No
Comorbidity	Diabetes	Diabetes Cirrhosis Alcoholism	Diabetes	Diabetes	No	Diabetes	Diabetes	Diabetes	Diabetes
Hemoptysis	Massive	Mild	Mild	N/A	Mild	No	No	Mild	Mild
Other symptoms	Fever Dry cough Dyspnea	Fever Productive cough Dyspnea	Fever Productive cough Dyspnea Chest pain	Productive cough Dyspnea Chest pain	Productive cough Dyspnea Chest pain	Fever Productive cough Dyspnea Chest pain	Fever Dry cough Chest pain	Fever Productive cough Dyspnea Chest pain	Fever Dry cough Dyspnea Chest pain
**Blood test results**									
HbA1C (4-6.4%)	6.2	**9.5**	**12.1**	N/A	N/A	**11.5**	6.2	**11.3**	**10.2**
WBC (4–10 G/L)	**11.5**	7.0	**24.3**	**32.4**	7.8	**12.8**	**11.0**	**20.0**	**15.1**
CRP (< 10 mg/L)	**23.8**	**38.1**	**333.4**	**149**	4.8	**140**	N/A	**173**	**186**
Hgb (120–180 g/L)	**100**	125	148	**110**	**116**	**7.9**	**10.2**	**8.9**	**9.3**
**Microbiological test results of sputum and blood samples**									
Bacterial and fungal cultures of sputum and blood samples^a^	Negative	Negative	Negative	Negative	*Aspergillus fumigatus* (sputum)	Negative	Negative	Negative	Filamentous fungi (sputum)
AFB smear of sputum	Negative	Negative	Negative	Negative	Negative	Negative	Negative	Negative	N/A
Xpert MTB/RIF of sputum	Negative	Negative	Negative	Negative	Negative	Negative	Negative	Negative	Negative

Abbreviations: Dx, diagnosis; ICU, intensive care unit; WBC, white blood cells; CRP, C-reactive protein; Hgb, hemoglobin; AFB: acid-fast bacillus; MTB/RIF, mycobacterium tuberculosis/rifampicin; N/A: not applicable. Abnormal values are highlighted in bold. ^a^Only the first culture sample was recorded

Table 2 summarizes the bronchoscopic and contrast-enhanced chest CT findings of nine patients. PAP could also manifest as external airway compression (case 1). None of the bronchoalveolar lavage samples yielded positive cultures for mucormycosis. Chest CT revealed bilateral pulmonary involvement in more than half of the cases. While most patients presented with a single PAP, one patient exhibited up to five, resulting in a total of 16 PAPs across nine patients (9 located in the right lung and 7 in the left). The round type accounted for the majority (10/16), with diameters ranging from 5 to 40 mm. The most frequent surrounding PAPs abnormalities were consolidation (9/9 cases) and necrosis (8/9 cases), whereas the reverse halo sign (RHS) and pleural effusion were uncommon. Although PAPs could extend into the bronchi and pleura, such invasions were infrequent. Importantly, all patients demonstrated pulmonary consolidation, and six out of nine exhibited RHS.

**Table 2:** Bronchoscopic and imaging characteristics of nine patients

Variables	Case 1	Case 2	Case 3	Case 4	Case 5	Case 6	Case 7	Case 8	Case 9
**Flexible bronchoscopy**									
Airway appearance	Mild upper tracheal stenosis Tumor obstructing right S8-S10 bronchi	Not performed	Not performed	Not performed	Blood at right S10 bronchus	Thick sputum obstructing right bronchus	Not performed	Pus and patchy pseudomembranes in bilateral bronchi	Left upper and lower bronchi stenosis
Bacterial culture	*S. aureus*				Negative	Negative		Negative	Negative
Fungal culture	Negative				Negative	Negative		Negative	Negative
AFB / Xpert MTB/RIF	Negative				Negative	N/A		Negative	Negative
**Chest computed tomography with contrast injection**									
Unilateral / Bilateral	Unilateral	Bilateral	Bilateral	Bilateral	Bilateral	Unilateral	Unilateral	Bilateral	Unilateral
Single / Multiple	Single	Multiple	Multiple	Multiple	Multiple	Single	Single	Multiple	Single
Number of PAPs	1	1	1	3	1	1	1	5	2
PAPs	Right S6, saccular PA, invading bronchial branch, 20 × 23 mm^2^ Surrounding lesions: consolidation, bronchiectasis	Right S3, round PA, 6 × 6 mm^2^ Surrounding lesions: consolidation, necrosis, RHS	Right S10, round PA, 29 × 31 mm^2^, invading bronchus and pleura Surrounding lesions: consolidation, necrosis, RHS	Left S6, round PA, 37 × 26 mm^2^; Two PA at left S10, saccular PA, 15 × 13 mm^2^ and 27 × 21 mm^2^, invading bronchial branch Surrounding lesions: consolidation, necrosis, RHS	Right S10, round PA, 5 × 5 mm^2^ Surrounding lesions: consolidation, necrosis	Right S9, irregular PA, 30 × 40 mm^2^ Surrounding lesions: consolidation, necrosis, cavitation	Right S5, saccular PA, invading bronchial branch, 5 × 10 mm^2^ Surrounding lesions: consolidation, necrosis, cavitation	Two PA at left S9, round PA, 5-6 × 8-10 mm^2^; Three PA at right S6-S8-S9, round PA, 6-8 × 8-10 mm^2^, invading pleura Surrounding lesions: consolidation, necrosis, cavitation	Left S2, round PA, 19 × 13 mm^2^; Left S2, irregular PA, 38 × 33 mm^2^ Surrounding lesions: consolidation, necrosis, cavitation
Pleural effusion	Yes	No	Yes	No	No	No	No	No	No
Nodule	No	4 nodules, tree-in-bud	7 nodules	20 nodules	No	No	No	2 nodules	No
Consolidation	Yes	Yes	Yes	Yes	Yes	Yes	Yes	Yes	Yes
GGO	No	No	Yes	Yes	No	No	Yes	No	Yes
RHS	No	Yes	Yes	Yes	No	Yes	Yes	Yes	No

Abbreviations: AFB: acid-fast bacillus; MTB/RIF, mycobacterium tuberculosis/rifampicin; PAP, pulmonary artery pseudoaneurysm; GGO, ground-glass opacity; PA: pseudoaneurysm; RHS: reverse halo sign; N/A: not applicable

Among the nine patients diagnosed with PM, CT-guided transthoracic lung biopsy and surgical resection were the most frequently employed methods for tissue sampling. Positive sputum cultures for Mucor species were obtained in only two patients, requiring repeated cultures as the initial specimens were negative, a not uncommon finding in the microbiological diagnosis of *Mucor* species. Four patients received conservative treatment for hemoptysis, but only one showed a favorable response. Surgical resection, either pneumonectomy or lobectomy, was performed in five cases, while other approaches included coil embolization and pulmonary artery embolization. Most patients were initially treated with amphotericin B and subsequently switched to oral triazoles. Three patients died, two of whom from fatal hemoptysis Table 3.

**Table 3:** Treatment and outcome of nine patients

Variables	Case 1	Case 2	Case 3	Case 4	Case 5	Case 6	Case 7	Case 8	Case 9
**Definitive diagnostic method for pulmonary mucormycosis**									
Diagnostic method	CT-guided transthoracic lung biopsy	CT-guided transthoracic lung biopsy	Right pneumonectomy	Right pneumonectomy	CT-guided transthoracic lung biopsy	Right middle and lower lobectomy	Right middle lobectomy Lichtheimia sp. positive in intraoperative pus sample	CT-guided transthoracic lung biopsy Mucor sp. positive in third sputum sample	Mucor sp. was positive in fourth sputum sample
**Treatment of hemoptysis**									
Conservative treatment	Unresponsive	Unresponsive	Not performed	N/A	Response	No hemoptysis	No hemoptysis	Unresponsive	Not performed
Other treatments	Pulmonary artery embolization	Pulmonary artery embolization	Emergency right pneumonectomy	Emergency right pneumonectomy	Elective right upper lobectomy	Elective right middle and lower lobectomy	Elective right middle lobectomy	No	Pulmonary artery embolization
**Antifungal treatment and outcome**									
Antifungal (including treatment at the primary care level)	AMB for 35 days, then switched to oral posaconazole	AMB for 40 days	AMB for 10 days, then switched to oral itraconazole	No	AMB for 15 days	AMB for 13 days	AMB for 14 days	AMB for 10 days	AMB 28 days, then switched to oral posaconazole
Outcome	Survival	Survival	Survival	In-hospital mortality	Survival	Survival	Survival	In-hospital mortality	In-hospital mortality
Progression of hemoptysis	Stabilization	Stabilization	Stabilization	N/A	Stabilization	No hemoptysis	No hemoptysis	Fatal hemoptysis	Fatal hemoptysis

Abbreviations: AMB, amphotericin B; CT, computed tomography; N/A: not applicable

## Discussion

Our cohort provides an overview of PAP in patients with PM diagnosed over a five-year period at the National Lung Hospital, Hanoi, Vietnam. Based on this study, we would like to highlight two key practice points. First, contrast-enhanced chest CT should be performed to screen for PAP in patients with confirmed PM and poorly controlled diabetes, regardless of the presence or absence of hemoptysis, given the prevalence of PAP among PM cases, with approximately 13% (9 out of 69 patients). Second, on top of its mechanism for PAP formation through infection-related vascular inflammation, *Mucor* species possesses angioinvasive properties that enable direct invasion of the vessel wall and subsequent development of PAP. Therefore, particular attention should be given to screening PAP in areas of pulmonary consolidation and/or necrosis.

In PAP, the arterial wall rupture allows blood to escape the vessel, forming a sac confined by adjacent tissue or thrombus instead of the normal three-layer wall [1, 2]. A retrospective study involving 2,782 patients admitted with hemoptysis over four years reported that PAP was detected by CT or DSA pulmonary angiography in 50 patients (1.8%) [3]. However, the frequency of PAP may rise to as high as 11% in patients undergoing bronchial angiography for hemoptysis [4]. The true prevalence of PAP remains uncertain, as most available studies are retrospective and primarily based on chest CT findings [3, 4, 17–19]. We believe the actual rate is underestimated, given that approximately 46% of PAPs are not reported on initial CT scans [17]. In our case series, the prevalence of PAPs among patients with PM was relatively high, at around 13%. PAP may arise from either congenital or acquired causes, among which infection and malignancy are the two most frequent etiologies, accounting for approximately 33–97% and 8–13% of cases, respectively [3, 17–19]. Currently reported infectious causes of PAP include infective endocarditis, pulmonary tuberculosis, pyogenic bacterial pneumonia (such as *S. aureus*, *S. pyogenes*, and *Klebsiella*), and indeed fungal infections (*Mucor*, *Aspergillus*, and *Candida*) [17, 20]. Among infection-related PAP, pulmonary tuberculosis is the predominant cause (60–79%), followed by necrotizing pneumonia (14–21%) and fungal pneumonia (12–21%) [3, 18, 19]. However, in the two available studies on PAP associated with fungal pneumonia, the causative fungal species were either not specified or reported exclusively as fungus balls [3, 19]. Consequently, PAP associated with PM has only been reported in isolated cases [7, 8, 15, 21, 22]. To our knowledge, this is the largest case series to date, in which all CT scans of patients diagnosed with PM were retrospectively scrutinized for PAP.

PM may lead to the development of PAP through two mechanisms. The first involves inflammation of the vascular wall adjacent to cavitary or necrotic pulmonary lesions, along with extension through septicemia [7, 20]. The second mechanism is the direct invasion and destruction of the vascular wall by Mucor species, leading to thrombosis and subsequent infarction [20, 23]. Both mechanisms contribute to progressive thinning of the arterial wall, ultimately resulting in the formation of PAP. PAP may remain asymptomatic or present only with minor hemoptysis; however, in severe cases, it can cause massive hemoptysis, respiratory failure, and rapid death [1, 2]. Studies have shown that PAP is asymptomatic in 3–21% of patients and manifests as massive hemoptysis in 13–28% [3, 17, 18]. Approximately 42% of PAPs eventually rupture, with a mortality risk of around 50% among these patients [3, 20]. Reported risk factors for rupture include earlier massive hemoptysis, large PAP diameter, cavitary lesions, and large cavity size [3]. Clinical scenarios in which PAP should be suspected include: (i) sudden massive hemoptysis, (ii) necrotizing pneumonia with pulmonary cavities, (iii) recurrent hemoptysis despite successful embolization of bronchial and non-bronchial arteries, (iv) normal findings on bronchial artery angiography, and (v) nodular or saccular contrast filling detected on enhanced CT scans [20]. We suggest that PM should be considered a risk factor for PAP and recommend that screening for PAP should be performed in patients diagnosed with PM, particularly in areas of pulmonary consolidation and/or cavitary necrosis.

Mucormycosis is caused by fungal species of the order Mucorales and can affect multiple organs, with PM representing the third most common site of involvement (20%), following cutaneous (22%) and rhino-orbito-cerebral (34%) [10]. Despite advances in diagnosis and treatment, both the incidence and mortality of mucormycosis have continued to rise in recent years [24]. PM carries a high mortality rate, ranging from 41% to 57%, underscoring the need for accurate diagnosis and timely treatment [12, 25]. Typical risk factors include immunosuppression due to hematological malignancies or solid-organ transplantation, as frequently reported in studies from developed countries [26, 27]. In contrast, poorly controlled diabetes, oral corticosteroid use, and other immunosuppressive therapies appear to be more common risk factors in reports from developing countries. An Indian study demonstrated that 91% of patients with PM had diabetes, the majority with poor glycemic control, while a study from China found diabetes in 71.7% of PM cases [25, 28]. Studies have shown that elevated serum glucose and diabetic ketoacidosis facilitate Mucor species proliferation, impair the phagocytic function of alveolar macrophages, and exacerbate tissue destruction mediated by fungal invasion [10, 11, 23]. PM may clinically resemble community-acquired pneumonia, presenting as fever, dyspnea, and cough; however, vascular invasion can lead to hemoptysis [11, 29]. The radiological hallmark often reported in PM is the RHS on CT scans. However, its prevalence varies widely from 19% to 95%, depending on patient characteristics such as leukocyte count, comorbidities, disease stage, and timing of imaging, which complicates its diagnostic value [11, 23]. PAP in the setting of PM is exceedingly uncommon; in one study involving 30 patients with PM, only a single case of PAP was identified [30]. Our case series highlights that PAP associated with PM predominantly occurs in patients with poorly controlled diabetes and most commonly presents with mild hemoptysis.

Histopathological examination and fungal culture of lower respiratory tract secretions remain the gold standard for diagnosing PM [31]. In patients with PM presenting with hemoptysis, obtaining sputum or bronchoalveolar lavage specimens may increase the risk of recurrent hemoptysis and respiratory failure compared with those without hemoptysis. Traditional fungal culture for Mucor yields positive results in only 15–25% of cases, leading to underdiagnosis [11, 32]. Although serum PCR assays have shown promise in recent studies, they have not yet been standardized or adopted as a diagnostic criterion [33]. In our study, 8 out of 9 patients were diagnosed with PM based on lung histopathology (4 from surgical resection and 4 from CT-guided biopsy), underscoring the challenges in establishing a definitive diagnosis, particularly in cases complicated by hemoptysis. In our practice, all patients with suspected PM undergo contrast-enhanced chest CT to evaluate for the presence of PAP before CT-guided biopsy. When PAP is identified, angiographic evaluation is considered, and pre-biopsy embolization is performed in selected patients, particularly those with active hemoptysis and unresponsive to conservative treatment (Patients 1 and 2), to mitigate the risk of bleeding. In patients with mild hemoptysis that responds to medical therapy (Patient 5), CT-guided biopsy may be performed once clinical stability is achieved. The procedure is meticulously planned to avoid the known vascular lesion, with the needle trajectory directed toward the cavity wall, consolidation, or necrotic areas suspicious of PM. In one case (Patient 8), although the biopsy was completed without procedure-related complications, the patient subsequently died due to disease progression and rupture of PAP, which was not attributable to the biopsy. A multidisciplinary approach and individualized risk-benefit assessment are essential to optimize procedural safety in this complex clinical setting.

Currently, limited research has addressed PAP associated with PM, and managements are mainly based on empirical treatment. The cornerstone of PM management includes intravenous antifungal therapy, optimization of underlying comorbidities, and surgical resection [11]. Liposomal amphotericin B remains the first-line agent, followed by oral isavuconazole or posaconazole in clinically stable patients [31, 34]. The most recent guidelines strongly recommend early and complete surgical intervention whenever possible, in addition to systemic antifungal therapy [34]. Endovascular management of PAP includes direct coil embolization of the pseudoaneurysm sac, covered stent placement, or embolization of the feeding pulmonary arterial branch [20, 35]. Nevertheless, these techniques may be insufficient when persistent bronchopulmonary shunts are present, as continued inflow from hypertrophied bronchial or non-bronchial systemic arteries can maintain perfusion of the pseudoaneurysm sac, a phenomenon commonly observed in infection-related PAP [20, 35]. Akis reported that 100% of PAP cases associated with pulmonary tuberculosis and 50% of those related to necrotizing pneumonia (predominantly in chronic inflammatory settings) demonstrated concomitant hypertrophy of bronchial or non-bronchial systemic arteries [36]. Similarly, Li found that 82.6% of infection-related PAP cases were accompanied by systemic-to-pulmonary arterial shunts [37]. These findings underscore the complex dual vascular supply frequently encountered in infectious PAP. Although none of the patients in our case series underwent additional bronchial artery evaluation, we suggest that comprehensive angiographic assessment, including both catheter-based pulmonary arteriography and selective bronchial/non-bronchial systemic artery angiography, should be considered [20]. This approach facilitates detection of vascular hypertrophy or systemic-to-pulmonary arterial shunting and allows for embolization of all significant feeding vessels, ideally during the same session as pulmonary artery embolization [20, 36]. Such a strategy may improve the effectiveness and durability of hemoptysis control in this challenging clinical context. Only about 55% of patients with PM are eligible for surgical intervention, among whom 82% survive, compared with 11% survival in those managed without surgery [38]. Severe comorbidities and patient refusal are the most common reasons for delaying surgery [38]. Patients with massive hemoptysis secondary to PAP and PM often have a poor postoperative prognosis due to the high risk of airway compromise [1, 35]. Emergency surgery in this setting is associated with substantial morbidity and mortality, with reported mortality rates exceeding 40% [19, 36]. In several tertiary centers, endovascular intervention is used as a bridging therapy to achieve hemostasis and stabilize the patient, followed by definitive pulmonary resection if the risk of recurrent hemorrhage persists [1]. Nevertheless, we suggest that surgery should remain the preferred therapeutic option in cases of PAP complicating PM. In our institution, patients undergoing surgical resection for PM with concomitant PAP receive intravenous amphotericin B for approximately 13–15 days, encompassing both preoperative and postoperative periods. Oral step-down therapy is not routinely administered when surgery is considered curative, defined by complete resection, clear surgical margins, absence of intraoperative spillage of infected parenchyma, and no fungal contamination of the pleural cavity (Patients 5, 6, and 7). As there is currently no established evidence regarding the optimal duration of perioperative antifungal therapy in surgically managed PM, treatment decisions are guided by institutional experience, multidisciplinary evaluation, and close postoperative follow-up.

This study has several limitations that should be acknowledged. First, its retrospective single-center design and the small number of patients with concomitant PM and PAP limit the statistical power and generalizability of the findings. Second, reliance on electronic medical records may have resulted in incomplete data capture and potential information bias. Third, outcomes were assessed only at hospital discharge, with no long-term follow-up available to evaluate recurrence or survival. Fourth, the limited sample size precluded comparative or multivariable analyses to identify independent prognostic factors. Finally, although PM was confirmed using defined diagnostic criteria, it was not feasible to systematically exclude all alternative causes of PAP, particularly rare etiologies such as Behçet’s disease or Hughes-Stovin syndrome, which should be considered when interpreting the results.

## Conclusion

In summary, this study provides the largest cohort to date of PAP associated with PM, offering valuable insights into its clinical features, diagnostic challenges, and outcomes. We emphasize that PAP should be systematically screened in patients with PM, particularly those with poorly controlled diabetes and in areas of pulmonary consolidation or cavitary necrosis. Early recognition and a combined strategy of antifungal therapy with timely surgical intervention may improve survival in this uncommon but life-threatening condition.

## Data Availability

The patient data supporting the findings of this study are available from the authors. Data are, however, available from the authors upon reasonable request.

## Abbreviations

AFB: Acid-fast bacillus
AMB: Amphotericin B
CRP: C-reactive protein
CT: Computed tomography
DSA: Digital subtraction angiography
EMR: Electronic medical record
GGO: Ground-glass opacity
ICU: Intensive care unit
PA: Pseudoaneurysm
PAP: Pulmonary artery pseudoaneurysm
PCR: Polymerase chain reaction
PM: Pulmonary mucormycosis
RHS: Reverse halo sign
WBC: White blood cells
